# HELLP (Hemolysis, Elevated Liver Enzymes, and Low Platelets)-Associated Subcapsular Hepatic Hematoma Presenting as Postoperative Hemorrhagic Shock: A Case Report

**DOI:** 10.7759/cureus.118908

**Published:** 2026-10-31

**Authors:** Julia Grossi Diaz, Karolyne Vale de Sá, Sarah Tanios Daneluzzi, Beatriz Saddi Nascimento Cavalcante Peres, Ana Clara Mazzetti, Beatriz Nascimento Motta Spaleta, Fernando Casella Szuster, Julia C Monteiro Klock

**Affiliations:** 1 Obstetrics and Gynecology, Universidade Santo Amaro, São Paulo, BRA; 2 Obstetrics and Gynecology, Universidade de São Paulo, São Paulo, BRA; 3 Research, Universidade de Mogi das Cruzes, São Paulo, BRA

**Keywords:** case report, damage control surgery, hellp syndrome, obstetric complications, preeclampsia, pregnancy, subcapsular hepatic hematoma

## Abstract

Subcapsular hepatic hematoma is a rare and life-threatening complication of HELLP (hemolysis, elevated liver enzymes, and low platelet count) syndrome associated with substantial maternal morbidity and mortality. We report the case of a 23-year-old primigravida at 27 weeks and 3 days of gestation who presented with severe frontal headache, nausea, vomiting, and hypertension and was initially diagnosed with preeclampsia with severe features. On hospital day three, clinical deterioration prompted emergency cesarean delivery, and postoperative laboratory findings confirmed HELLP syndrome. On the first postoperative day, the patient developed hypovolemic shock and signs of acute abdomen. Point-of-care ultrasonography revealed free intra-abdominal fluid, prompting urgent surgical re-exploration. An extensive subcapsular hepatic hematoma with active bleeding was identified, and damage control surgery with hepatic packing and temporary abdominal closure was performed. After 48 hours of clinical stabilization, planned re-exploration with pack removal and definitive abdominal wall closure was completed. Liver enzymes subsequently showed progressive normalization through hospital discharge. This case highlights the importance of recognizing hepatic complications of HELLP syndrome in patients with abrupt postoperative deterioration and demonstrates the role of coordinated multidisciplinary management in severe hemorrhagic presentations. In hemodynamically unstable patients with evidence of ongoing intra-abdominal hemorrhage, the absence of definitive cross-sectional imaging may not preclude urgent surgical exploration.

## Introduction

HELLP syndrome, an acronym for hemolysis, elevated liver enzymes, and low platelet count, is a severe microangiopathic complication of pregnancy, typically arising in the context of preeclampsia or eclampsia [1]. Diagnosis is based on laboratory criteria established by the American College of Obstetricians and Gynecologists (ACOG): lactate dehydrogenase (LDH) ≥ 600 IU/L, AST and ALT levels more than twice the upper limit of normal, and platelet count < 100,000/µL [2].

Among the complications of HELLP syndrome, subcapsular hepatic hematoma is a rare but potentially fatal manifestation, with an estimated incidence of 1 in 45,000 to 1 in 225,000 pregnancies [3]. Maternal mortality due to HELLP syndrome has been reported to range from 1% to 30% [4]. When complicated by subcapsular hepatic hematoma, maternal mortality was reported to be as high as 59%. However, with advances in early diagnosis and multidisciplinary management, the mortality rate has declined to 10%-15% [5]. Subcapsular hepatic hematoma occurs most commonly in the third trimester but may also present during the postpartum period [3]. Previous reports have described both conservative and surgical management strategies for HELLP-associated subcapsular hepatic hematoma. However, reports integrating detailed intraoperative findings, staged damage-control management, and serial biochemical evolution remain limited [5-11].

We report a case of HELLP-associated subcapsular hepatic hematoma presenting with postoperative hemorrhagic shock and requiring damage control surgery with hepatic packing and temporary abdominal closure. The case provides a detailed clinical timeline from maternal deterioration and intraoperative diagnosis through staged surgical management and biochemical recovery, highlighting practical considerations in the recognition and management of this rare obstetric emergency.

This work was previously presented as an e-poster at the 62nd Brazilian Congress of Gynecology and Obstetrics (CBGO), organized by the Brazilian Federation of Gynecology and Obstetrics Associations (FEBRASGO) and held at Riocentro, Rio de Janeiro, Brazil, from May 14 to 17, 2025.

## Case presentation

A 23-year-old primigravida at 27 weeks and 3 days of gestation presented to a public emergency department with a four-day history of severe bilateral frontal headache, photophobia, phonophobia, nausea, and vomiting, along with elevated blood pressure measured at home. She had no relevant family or past medical history beyond migraine, which had worsened during pregnancy. Her prenatal care had been uneventful until this presentation, with no previously documented hypertensive episodes.

On examination, her blood pressure was 170 × 102 mmHg, and she was normocardic and afebrile. The fundal height was 26 cm, the fetal heart rate was 138 bpm, and the uterine tone was normal, with no contractions. Bilateral lower-limb edema was graded as 3+/4+. Initial laboratory testing showed mild hepatic enzyme elevation, renal dysfunction, hyperuricemia, and significant proteinuria (Table 1, column 3).

**Table 1:** Serial laboratory findings during hospitalization ALT: Alanine aminotransferase; AST: Aspartate aminotransferase; LDH: Lactate dehydrogenase; PO: Postoperative; INR: International normalized ratio; “-” indicates that a value was not available for that time point.

Laboratory Test	Reference Range	Day 0 Admission	Day 1	Day 3 Pre-delivery Deterioration	Day 3 Immediate Postoperative	Day 4 PO Day 1	Day 5 PO Day 2	Day 6 PO Day 3	Day 14 PO Day 11	Day 18 Discharge
Platelet count (x10³/µL)	150-450	222	227	134	56	62	62	96	497	654
AST (U/L)	<35	43	32	45	153	554	620	243	41	43
ALT (U/L)	<33	45	35	50	109	415	531	356	51	43
LDH (U/L)	120-250	296	300	285	1.046	1.356	1.347	486	352	343
Creatinine (mg/dL)	0.6-1.1	1.3	1.1	1.2	1.2	1.2	-	0.7	0.5	-
Urea (mg/dL)	15-45	58.4	38.8	68.6	69.3	86.4	-	89	20.7	-
Uric acid (mg/dL)	2.6-6.0	8.2	9.6	10.8	10.8	-	-	-	3.5	-
Total bilirubin (mg/dL)	0.3-1.2	0.3	0.4	0.4	4.6	1.6	-	0.7	0.5	0.4
Hemoglobin (g/dL)	12.0-16.0	13.2	14.2	11.5	10.6	7.6	7.6	6.4	7.4	8.2
Hematocrit (%)	36-46	36.6	39.4	31.8	29.4	21.6	21.6	18.2	22.7	25.1
INR	0.9-1.2	-	-	0.8	0.8	0.93	1.21	1.08	1.04	-
Leukocyte count (mm³)	4,000-11,000	10,400	7,600	4,000	24,200	19,100	-	24,900	12,300	9,200
Urinary protein	Negative	3+	-	-	-	-	-	-	-	-

Obstetric Doppler ultrasound revealed normal amniotic fluid (deepest vertical pocket, 4.3 cm), an estimated fetal weight of 791 g (2nd percentile by Hadlock), consistent with fetal growth restriction, and no documented velocimetric abnormalities, as shown in Figure 1. A diagnosis of preeclampsia with severe features was established, and the patient was hospitalized for magnesium sulfate therapy and maternal-fetal surveillance.

**Figure 1:**
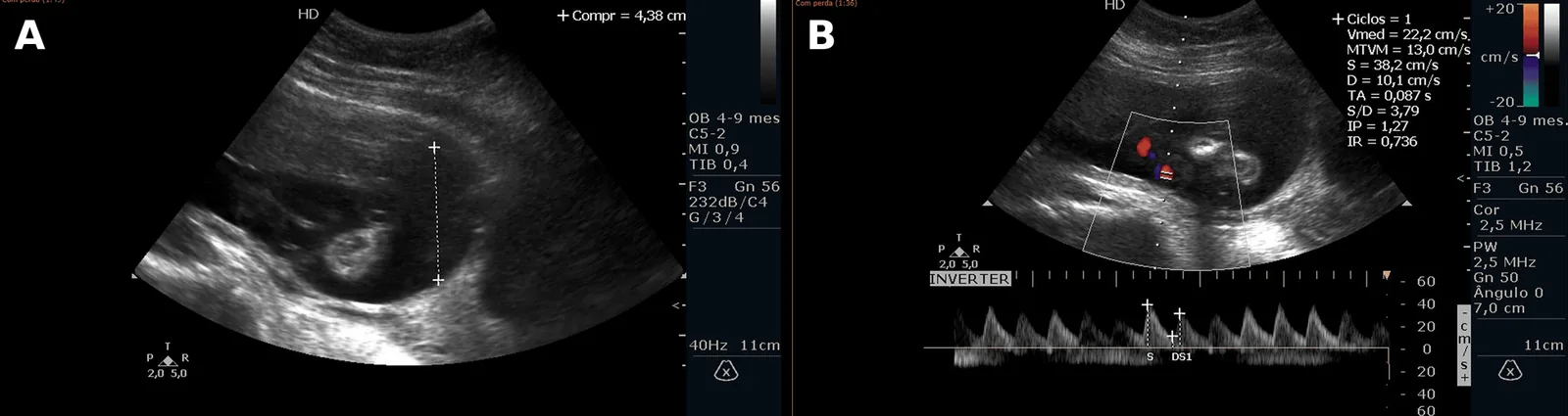
Obstetric ultrasound and Doppler assessment at admission (A) Measurement of the deepest vertical pocket of amniotic fluid, approximately 4.3 cm. (B) Color and spectral Doppler velocimetry obtained during the initial obstetric evaluation, demonstrating no velocimetric abnormalities.

On hospital day 3, the patient developed a documented febrile episode associated with tachycardia, a new hypertensive crisis, an initial decrease in leukocyte count, and thrombocytopenia (Table 1, column 5). Given the acute maternal deterioration and concern for possible sepsis, a sepsis protocol was initiated, blood cultures were obtained, and empiric broad-spectrum antibiotic therapy was started. Magnesium sulfate therapy was reinitiated, and emergency cesarean delivery was indicated because of the rapidly worsening maternal condition. The available aerobic and anaerobic blood culture reports subsequently showed no bacterial growth, and no definite infectious focus was identified. Intraoperatively, diffuse and difficult-to-control hemorrhage was noted. Intravenous tranexamic acid was administered, and a pelvic drain was placed. A live female neonate weighing 700 g was delivered, with Apgar scores of 5, 8, and 9 at 1, 5, and 10 minutes, respectively [12].

In the immediate postoperative period, laboratory findings confirmed HELLP syndrome, with a marked decrease in platelet count and concurrent elevations in hepatic enzymes and LDH (Table 1, column 6). Given the diffuse intraoperative bleeding and marked thrombocytopenia, an associated coagulopathic process was suspected. Serial INR measurements were subsequently obtained and remained only mildly altered, with a peak value of 1.21 (Table 1, row 12). The patient was admitted to the intensive care unit (ICU). On the first postoperative day, she deteriorated into hypovolemic shock, requiring vasopressor support and mechanical ventilation. The pelvic drain produced more than 1,000 mL of bloody output within 12 hours. Point-of-care ultrasound (FAST) revealed free intra-abdominal fluid, raising suspicion of intra-abdominal hemorrhage.

Urgent surgical re-exploration was performed jointly by the obstetrics and general surgery teams through a midline laparotomy. The uterus was well contracted, and the hysterotomy closure was intact. An extensive subcapsular hepatic hematoma associated with diffuse active bleeding was identified. Damage control surgery was conducted, consisting of hepatic packing with gauze compresses and temporary abdominal closure, as shown in Figure 2. The patient returned to the ICU under sedation, mechanical ventilation, and vasopressor support. At this stage, hepatic biochemical markers showed further deterioration, with a pronounced increase in liver enzyme and LDH levels on postoperative day 2 (Table 1, column 8).

**Figure 2:**
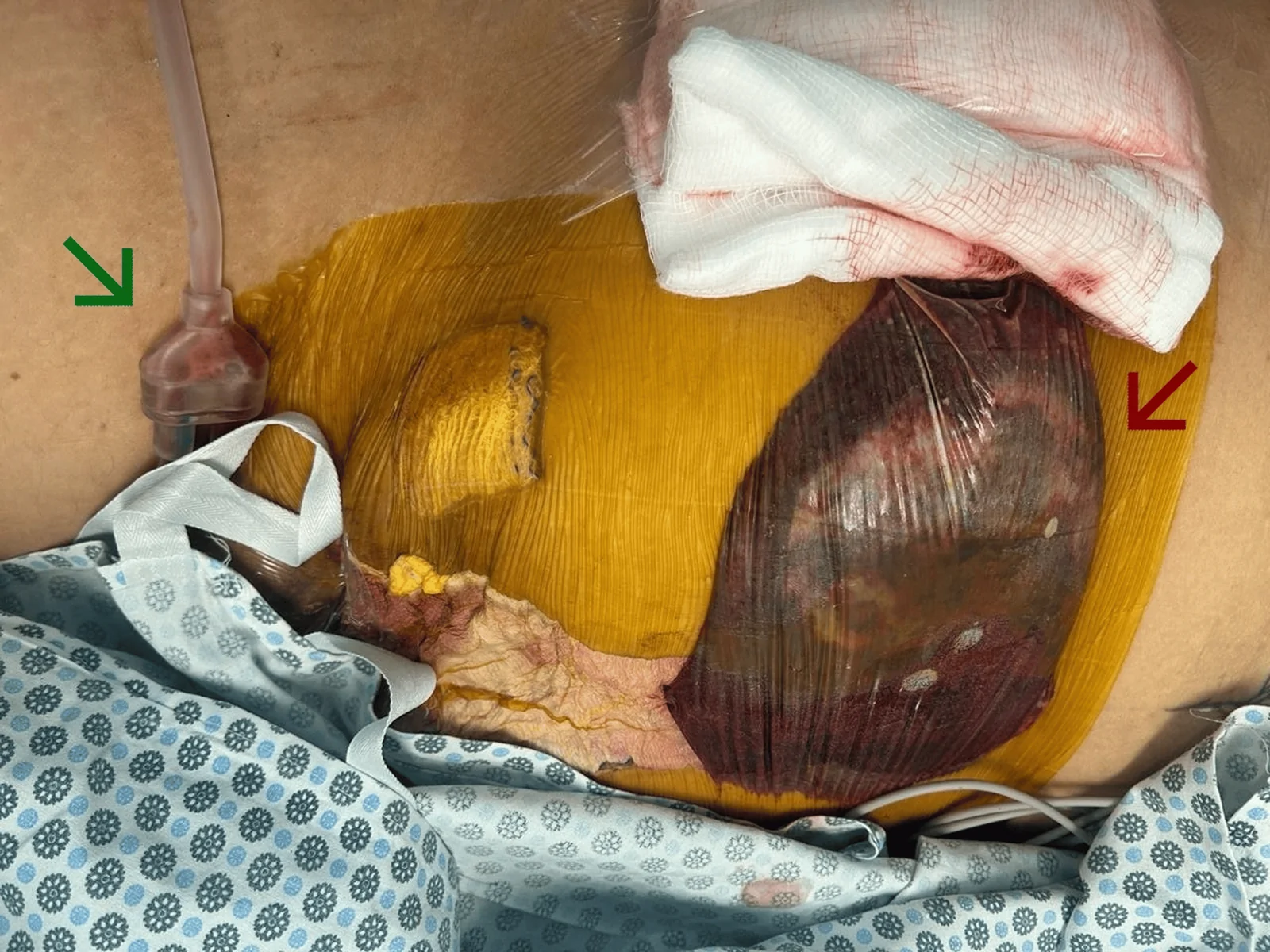
Temporary abdominal closure after damage control surgery for HELLP-associated subcapsular hepatic hematoma Hepatic packing with gauze compresses is visible (red arrow), and a pelvic drain is in situ (green arrow). Cranial is to the right, and caudal is to the left of the image. The abdomen remained open for 48 hours until clinical stabilization allowed definitive abdominal closure. HELLP: Hemolysis, elevated liver enzymes, low platelets.

During this period, resuscitative management included intravenous fluids, vasopressor support, and transfusion of two units of packed red blood cells and seven units of platelets. After 48 hours, with evidence of clinical stabilization, successful weaning from vasopressors, and lactate levels remaining ≤ 2 mmol/L, definitive surgical revision was performed. The packs were removed, hemostasis was confirmed, and the abdominal wall was closed. A nasogastric tube and a Portovac drain in the right flank were maintained. Progressive improvement in liver enzyme levels was documented over the subsequent days, with AST and ALT reaching near-normal values by postoperative day 11 (Table 1, column 10), as illustrated in Figure 3.

**Figure 3:**
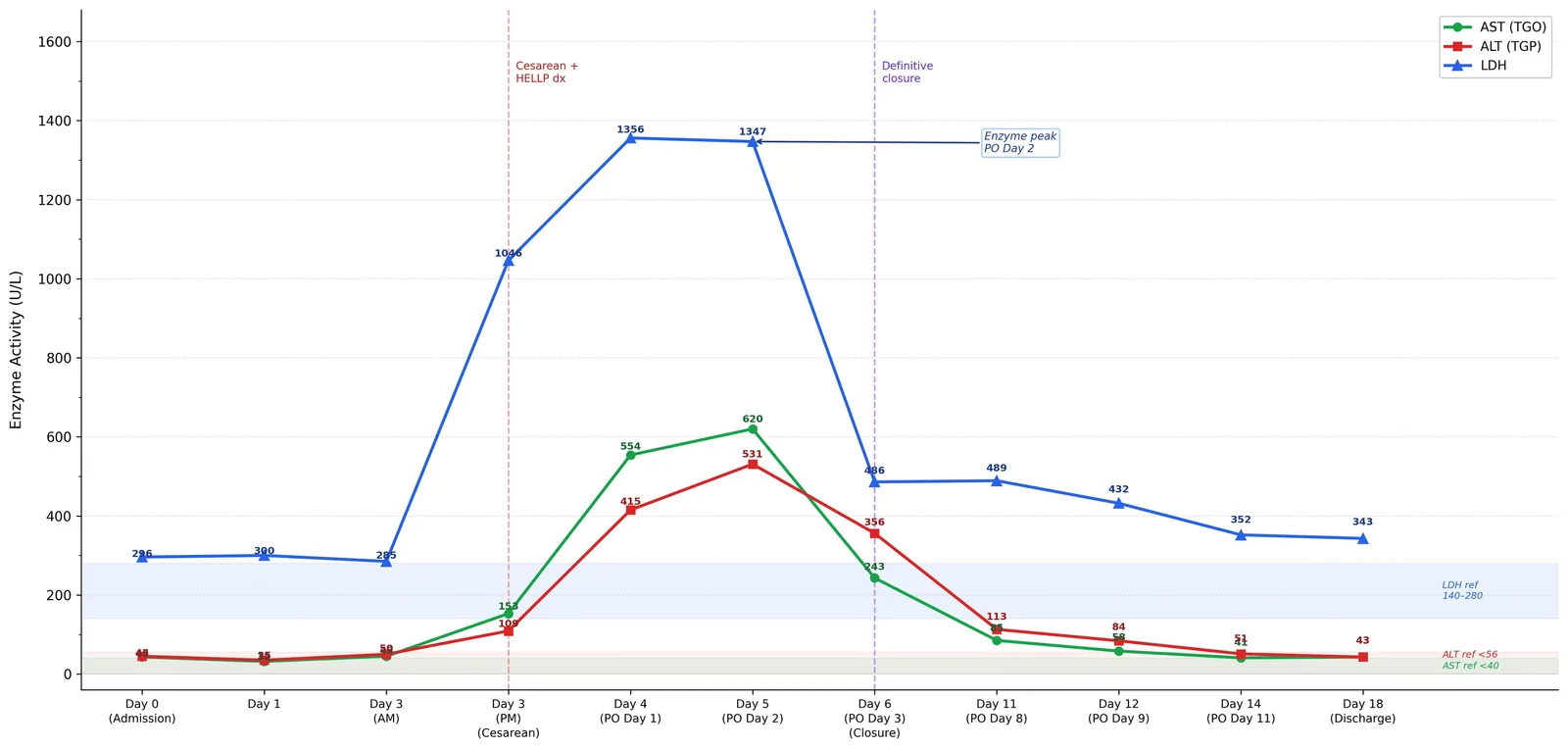
Serum liver enzyme trends during hospitalization demonstrating progressive normalization following surgical management Shaded bands indicate normal reference ranges. Dashed vertical lines indicate key surgical events: cesarean delivery (Day 3) and definitive abdominal closure (PO Day 3). ALT: Alanine aminotransferase; AST: Aspartate aminotransferase; LDH: Lactate dehydrogenase; HELLP: Hemolysis, elevated liver enzymes, low platelets.

The patient was transferred to the ward and subsequently discharged in good clinical condition on hospital day 18, with continued biochemical and hematologic recovery (Table 1, column 11). The complete clinical course, including key events, interventions, and laboratory findings, is summarized in Table 2.

**Table 2:** Clinical timeline: key events and interventions D: Hospital day from admission; PO: Postoperative day from cesarean section. ALT: Alanine aminotransferase; AST: Aspartate aminotransferase; BP: Blood pressure; Cr: Creatinine; DCS: Damage control surgery; FAST: Focused assessment with sonography for trauma; Hb: Hemoglobin; HELLP: Hemolysis, elevated liver enzymes, low platelets; LDH: Lactate dehydrogenase; MgSO₄: Magnesium sulfate; PLT: Platelets.

Phase	Hospital Day	Clinical Events	Key Interventions
1. Admission and surveillance	D0–D2	Severe fronto-temporal headache, photophobia, phonophobia, nausea, and vomiting	MgSO₄ loading + 24-h maintenance
		BP 170/102 mmHg and 3+/4+ lower-limb edema	Antihypertensive therapy
		3+ proteinuria	Maternal-fetal surveillance
		Working diagnosis: preeclampsia with severe features	
2. Maternal deterioration and delivery	D3	Fever, recurrent hypertensive crisis, and tachycardia	MgSO₄ reloaded
		Declining leukocyte and platelet counts	Ceftriaxone + metronidazole (sepsis protocol)
		Emergency cesarean section	Emergency cesarean delivery
		Live female neonate, 700 g, Apgar 5/8/9	IV tranexamic acid
		Diffuse intraoperative hemorrhage	Pelvic drain
		HELLP syndrome confirmed postoperatively	
3. Postoperative deterioration	D4 (PO D1)	Hypovolemic shock (peritoneal signs)	Norepinephrine + mechanical ventilation
		Pelvic drain output > 1000 mL/12 h	Joint laparotomy by the obstetrics and general surgery teams
		FAST: free intra-abdominal fluid	Damage control surgery: hepatic packing + temporary abdominal closure
		Surgical re-exploration: extensive subcapsular hepatic hematoma with active bleeding	
4. Definitive repair	D6 (PO D3)	Clinical stabilization	Abdominal wall closure
		Vasopressor weaning	Nasogastric tube
		Definitive abdominal wall closure	Right-flank Portovac drain
5. Recovery and discharge	D11–D18 (PO D8–D15)	Transfer to ward	Outpatient follow-up arranged
		Progressive normalization of liver enzymes and platelet recovery	
		Hospital discharge on D18 in good clinical condition	

Written informed consent for publication of this case report, including the patient's clinical data and accompanying images, was obtained from the patient. The study was approved by the Research Ethics Committee of Universidade Santo Amaro (Approval No. 7.281.843; CAAE 85301824.0.0000.0081).

## Discussion

HELLP syndrome is a severe obstetric emergency characterized by systemic endothelial injury, coagulation activation, and progressive organ dysfunction. The exaggerated inflammatory response leads to placental ischemia and maternal vascular endothelial damage, promoting platelet adhesion, coagulation cascade activation, and complement system dysregulation, resulting in microangiopathic hemolytic anemia and thrombocytopenia. Fibrin deposition in the hepatic microvasculature obstructs sinusoidal flow, causing hepatocellular injury and transaminase elevation [13,14].

Subcapsular hepatic hematoma represents one of the most severe complications of HELLP syndrome. In a 25-year retrospective series of 23 cases, hemorrhagic shock and capsular rupture were independently associated with maternal mortality, with odds ratios of 36.0 and 10.5, respectively [7]. Clinical manifestations are frequently nonspecific and may include right upper quadrant or epigastric pain, hemoperitoneum, hemodynamic instability, and rapid deterioration [3,6]. In some cases, the hepatic source of bleeding is identified only during surgical exploration [3,5,9]. In the present case, the source was similarly recognized during urgent postoperative re-exploration.

A noteworthy laboratory finding in the present case was the delayed peak in hepatic biochemical markers on postoperative day 2 (Table 1, column 8), which occurred two days after cesarean delivery and one day after damage control surgery and exceeded the values observed at the time of HELLP syndrome diagnosis (Table 1, column 6). This delayed biochemical peak was likely multifactorial, reflecting the underlying hepatic injury associated with HELLP syndrome and the subcapsular hepatic hematoma, with additional contributions from hepatic hypoperfusion during hemorrhagic shock and possibly local compression related to hepatic packing. Notably, aminotransferase levels at the time of HELLP syndrome diagnosis were only moderately elevated. Previous studies have shown that the magnitude of liver enzyme abnormalities does not reliably correlate with the presence of hepatic imaging abnormalities in HELLP syndrome, whereas more severe thrombocytopenia is associated with abnormal hepatic imaging findings [15].

Imaging with ultrasonography, computed tomography, or magnetic resonance imaging can support presurgical diagnosis. However, in hemodynamically unstable patients, point-of-care ultrasound (FAST) may provide rapid evidence of intra-abdominal free fluid when transport for definitive cross-sectional imaging is unsafe, as demonstrated in this case [16].

Management depends on the patient’s hemodynamic status. Stable patients may be managed conservatively with volume resuscitation, blood product transfusion, and close monitoring. When instability persists, as in the present case, surgical intervention is recommended [16]. Options include hepatic artery embolization, surgical tamponade, ligation, or hepatic resection, depending on severity [17]. Damage control surgery, comprising hepatic packing and temporary abdominal closure, was chosen in this case given the extent of ongoing hemorrhage, hemodynamic instability, and severe thrombocytopenia, allowing physiological stabilization before definitive repair.

Early coordination between obstetric and general surgery teams is essential when intra-abdominal hemorrhage is suspected, particularly when the bleeding source may be extrauterine. Predefined criteria for multidisciplinary surgical assessment may facilitate timely escalation of care in rapidly deteriorating patients. The progressive normalization of liver enzymes documented in this case, with AST and ALT levels returning to near-normal values by postoperative day 11, illustrates the potential for recovery of hepatic injury following successful hemorrhage control.

This report has limitations inherent to its retrospective single-case design. Definitive cross-sectional imaging was not obtained before surgical re-exploration because of the patient's hemodynamic instability, limiting radiological characterization of the hematoma before operative management. In addition, the findings from a single clinical course cannot establish the superiority of damage control surgery over other management strategies. Nevertheless, the detailed perioperative timeline, operative findings, and serial biochemical data provide a clinically informative account of staged surgical management in this uncommon obstetric emergency.

## Conclusions

Subcapsular hepatic hematoma, although rare, represents a potentially fatal complication of HELLP syndrome. In hemodynamically unstable patients, prompt multidisciplinary surgical intervention can be life-saving. Damage control surgery, when applied promptly and in a multidisciplinary setting, may allow favorable maternal recovery even in severe cases. This case underscores the importance of early recognition of hepatic complications in HELLP syndrome, particularly in the setting of abrupt postoperative deterioration, and highlights the critical role of team-based care, sequential biochemical monitoring, and emergency surgical intervention. The complete perioperative course documented here, including serial laboratory normalization, may serve as a practical reference for physicians managing this rare but life-threatening complication. The development and implementation of structured institutional protocols for hepatic complications of HELLP syndrome may help reduce maternal morbidity and mortality associated with this condition.
